# CTLA4-Ig (abatacept) therapy modulates T cell effector functions in autoantibody-positive rheumatoid arthritis patients

**DOI:** 10.1186/1471-2172-14-34

**Published:** 2013-08-05

**Authors:** Jennifer Pieper, Jessica Herrath, Sukanya Raghavan, Khalid Muhammad, Ronald van Vollenhoven, Vivianne Malmström

**Affiliations:** 1 Rheumatology Unit, Department of Medicine at Karolinska University Hospital, Karolinska Institute, Solna, Stockholm, Sweden; 2 Unit for Clinical Therapy Research, Inflammatory Diseases, Karolinska Institute, Solna, Stockholm, Sweden

**Keywords:** Rheumatoid arthritis, Autoimmunity, T lymphocyte, Cytokines, Regulatory T cells, Abatacept, ACPA

## Abstract

**Background:**

Rheumatoid arthritis is a chronic inflammatory disease with a strong MHC class II component and where many patients develop characteristic autoantibodies towards the noncoding amino acid citrulline. Such anti-citrullinated protein antibodies (ACPA) have recently been put forward as an independent predictive factor for treatment response by co-stimulation blockade by CTLA4-Ig (abatacept). We have performed a mechanism of action study to dissect T cell functionality in RA patients with long-standing disease undergoing abatacept treatment and the influence of ACPA status.

**Results:**

Peripheral blood samples were collected from RA patients as they started CTLA4-Ig treatment and 3 and 6 months later. A general decrease of regulatory T cell subsets was observed in the cohort. Additionally within the ACPA-positive group significant down-regulation of all key T cell effector subsets including Th1, Th2, and Th17 was observed by analyzing cytokines by intracellular flow cytometry and in cell culture supernatants.

RA synovial fluid samples were cultured in vitro in the presence or absence of CTLA4-Ig (abatacept). T cell cytokine production was diminished, but without increasing the functional capacity of CD4+CD25hi regulatory T cells as previously demonstrated in the context of TNF-blockade and anti-IL6R therapy.

**Conclusions:**

Our immunological study of T cell functionality in RA patients, both ACPA-positive and ACPA-negative, starting biological therapy with the co-stimulation blockade abatacept (CTLA4-Ig) supports the recently published registry study implicating ACPA seropositivity as an independent predictive factor to treatment response as we observed the most striking effect on T cell subset modulation in ACPA-positive patients. These data further support the notion of RA as a disease with several sub-entities, where the ACPA-positive fraction represents a classical HLA-associated autoimmune disorder while ACPA-negative patients may have other driving forces apart from classical adaptive immune responses.

## Background

Rheumatoid arthritis (RA) is a systemic inflammatory disorder characterized by chronic joint inflammation and continuous cell infiltration into the synovium. The presence of anti-citrullinated protein antibodies (ACPA) in a large subset of patients supports the notion of an autoimmune etiology and is tightly associated with the HLA-DR shared epitope alleles, which suggests that CD4+ T cells are important [1]. Indeed, CD4+ T cells are abundant in both synovial tissue and in synovial fluid. Furthermore, ACPA-positive RA patients develop generally a more aggressive disease than ACPA-negative patients [2,3].

The role of CD4+ T cells in RA pathophysiology may be mediated through Th1 effector functions, mainly IFN-γ secretion [4], Th17 activity or induction of ACPA [5,6], leading finally to bone and cartilage destruction. It has also been suggested that regulatory T cell (Treg) function may be impaired in RA [7]. Tregs represent a crucial T cell subset in the maintenance of immune homeostasis and are significantly enriched in the synovial fluid of RA patients [8,9].

Commonly used biological therapies for RA target mainly cytokine pathways, with the exception of the B cell depleting agent rituximab and abatacept, a chimeric CTLA4 and IgG Fc fusion protein modulating T cell activation. Abatacept is believed to work by blocking CD28 costimulation and thereby interfering with T cell-APC interaction and limiting T cell activation. Use of abatacept is associated with reduction in joint inflammation, pain and joint damage in patients with active RA [10]. Recently it has been reported that abatacept shows a better clinical response in ACPA-positive as compared to ACPA-negative patients [11]. Many lines of evidence suggest that the CD4+ T cell compartment is more active in ACPA-positive as compared to ACPA-negative RA.

In this study we compared T cell functionality in the context of ACPA status in patients before and after T cell costimulation blockade by abatacept. Our data demonstrate that abatacept therapy significantly reduces circulating Treg frequencies, as well as effector cytokine output from Th1, Th2 and Th17 cells in ACPA-positive patients, but not in ACPA-negative patients. We further validated these findings in synovial fluid cell cultures where pharmacological doses of abatacept were added. We conclude that abatacept interferes with the full range of T cell subsets, especially T cells in ACPA-positive patients.

## Results

### Unchanged levels of autoantibodies in abatacept-treated patients

For this study 33 patients starting on abatacept therapy were included and peripheral blood was collected at baseline and after 3 and 6 months. It has recently been reported that treatment with abatacept shows better results in ACPA-positive individuals, and we therefore examined both the ACPA status of our patient cohort at baseline and investigated changes in ACPA levels from baseline to the 6 months follow-up.

**Figure 1:**
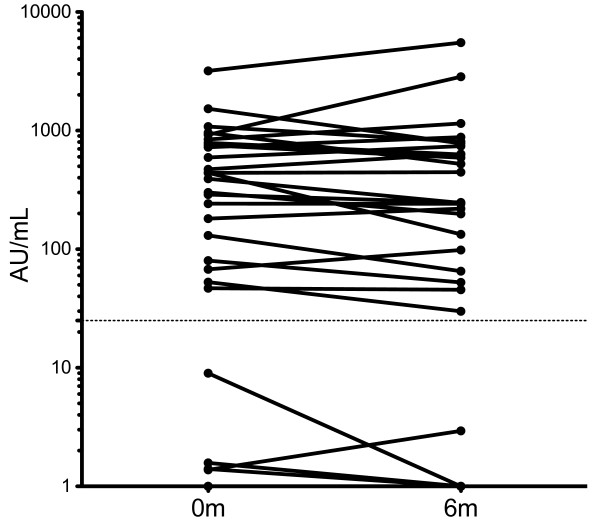
**ACPA level at baseline and 6 months following abatacept treatment.** ACPA level was measured in serum of 33 RA patients undergoing abatacept therapy at baseline and 6 months following treatment. Samples ≥ 25 AU/mL are defined as positive.

In the cohort, 23 of 33 investigated patients had serum antibodies against citrullinated proteins (Figure 1), but we did not observe any significant changes in the ACPA levels during the study period, nor did the ACPA status predict clinical outcome; however this study was not intended and powered to adequately address this issue. Our aim instead was to investigate the adaptive immune function.

### Diminished T cell effector functions in patients treated with abatacept

We started by investigating the most relevant cytokines in the context of RA, namely IFN-γ, TNF and IL-17, as they are all implicated in disease pathogenesis and are central in Th1 and Th17 function.

**Figure 2:**
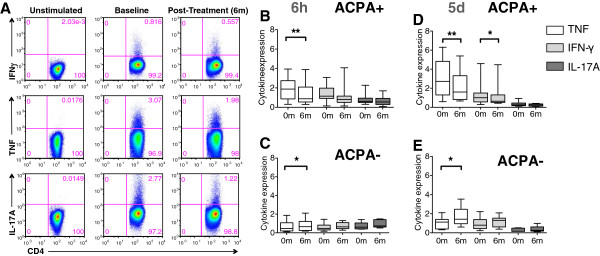
**Flow cytometry staining for intracellular cytokines TNF, IFN-γ, IL-17A.** PBMCs were stimulated in vitro with α-CD3 for 6 h or 5 days and intracellular cytokine staining was performed for IFN-γ, TNF and IL-17A. The plotted cells are gated as CD14-CD3+CD4+CD28+ T cells. **(A)** A representative FACS staining. Left row: Unstimulated cells. Subsequent rows: α-CD3 stimulation at baseline and 6 months following treatment. **(B**-**E)** Intracellular cytokine stainings. **(B)** ACPA-positive patients, 6h stimulation (n=12). **(C)** ACPA-negative patients, 6h stimulation (n=7). **(D)** ACPA-positive patients, 5days stimulation (n=12). **(E)** ACPA-negative patients, 5days stimulation (n=6).

At baseline and six months following abatacept therapy, PBMCs from 19 patients were utilized for polyclonal T cell stimulation for both 6 hrs and 5 days, and subsequently intracellular cytokine stainings were performed. By this approach, each individual is its own control. Representative flow cytometry stainings are depicted in Figure 2A. While unstimulated cells did not produce cytokines, robust production was observed following polyclonal stimulation (Figure 2A).

Our data show that T cells of the Th1 subset from ACPA-positive patients are clearly affected by abatacept, as both TNF and IFN-γ production by CD4+ T cells were significantly decreased at the day 5 read-out (Figure 2D). Only minor secretion of IL-17A could be observed after polyclonal stimulation, but we still saw a tendency in diminished IL-17A production after treatment (Figure 2B and D). In contrast, cells from ACPA-negative patients displayed the opposite pattern with increased cytokine production of TNF, IFN-γ and IL-17A (Figure 2C and E).

Our multiparameter flow approach allows a comparison of the different cytokines within each patient sample. To this end we calculated ratios of each cytokine and sample by comparing 6 months with baseline. Again, all ACPA-negative patients showed higher ratios (>1), i.e. increased expression while the ACPA-positive patients mostly displayed reduced cytokine output (<1) (data not shown). From these ratios we further determined the spearman’s rank correlation coefficients for the different cytokine combinations. A significant correlation between all examined cytokines (IFN-γ/TNF: r=0.651, p=0.003; TNF/IL-17A: r=0.688, p=0.002; IL-17A/IFN-γ: r=0.558, p=0.016) was detected demonstrating that the effect of CTLA-Ig was general rather than biased.

### Abatacept modulates key cytokines influencing different T helper subsets

Abatacept limits the immune response by binding to CD80 and 86 on antigen-presenting cells (APCs). Hence, we investigated the immune-modulatory effect of abatacept in the cell culture supernatants of polyclonal stimulated PBMCs from 17 abatacept-treated patients by luminex and a panel of 15 cytokines. We focused on cytokines relevant for the Th1, Th2 and Th17 subsets.

**Figure 3:**
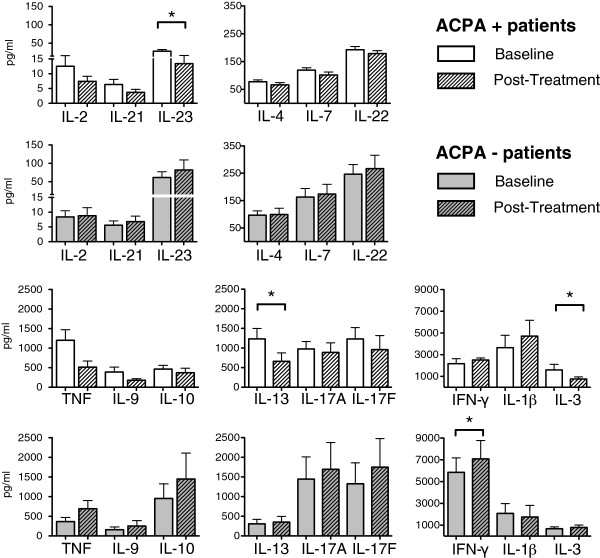
**Cytokines in cell culture supernatants.** PBMCs were stimulated in vitro with α-CD3 for 5 days and supernatants were taken and examined by the luminex method for 15 cytokines. Cytokine levels of 17 patients at baseline (non hatched bars) and post-treatment (hatched bars). White bars represent ACPA-positive patients (n=10), whereas grey bars represent ACPA-negative patients (n=7).

In ACPA-positive patients IL-3, IL-13 and IL-23 were significantly diminished after abatacept treatment (Figure 3). IL-2, -4, -7, -9, -10, -17A, -17F, -21, -22 and TNF were also diminished, but the change did not reach statistical significance. IL-1β levels tended to increase after abatacept treatment in this patient group.

For ACPA-negative patients IFN-γ was significantly increased (Figure 3). Many other examined cytokines were also increased but without reaching statistical significance.

### Abatacept reduces regulatory T cell frequencies in vivo

Next we investigated the phenotype of Tregs in patients undergoing abatacept therapy.

Polychromatic flow cytometry was performed on peripheral blood samples of 12 RA patients taken at initiation of treatment (0 months) and 3 months post-treatment. In addition to FOXP3, we also investigated Helios, CD39 and CTLA4 as they have been implicated in Treg function in RA [12-16], as well as CD45RA to also address naïve Tregs.

**Figure 4:**
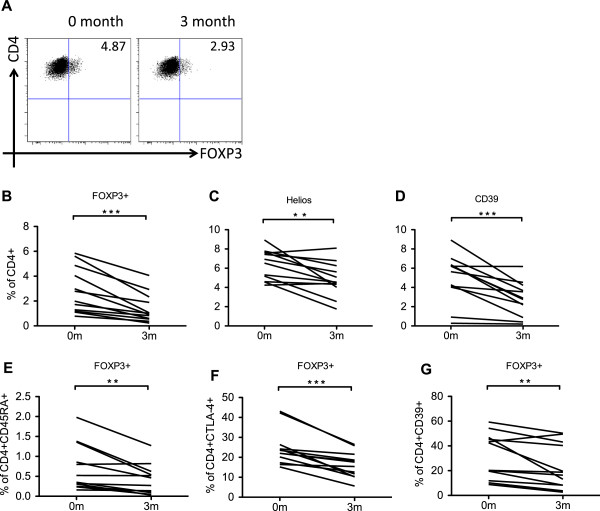
**Treg frequency is diminished.** PBMCs from baseline and 3 months following treatment (n=12) were obtained and multicolor flow cytometry was performed. A general reduction in frequency of several Treg associated markers could be seen **(A**-**G)**. **(A)** The graph depicts a representative staining of CD4+FOXP3+ Treg before and after treatment. **(B)** The graph displays CD4+FOXP3+ Treg, **(C)** Helios+ T cells, **(D)** CD4+CD39+ T cells, **(E)** CD45RA+FOXP3+ Treg, **(F)** CTLA4+ FOXP3+Treg, and **(G)** CD39+FOXP3+ Treg.

A representative staining of FOXP3 expression before and after therapy is depicted in Figure 4A. A significant reduction in the frequency of FOXP3+ Tregs, Helios+ and CD39+ T cells was observed at 3 months as compared to baseline (Figure 4B-D). To dissect this further, we also analyzed the proportion of naïve thymus-derived Tregs based on CD45RA expression in conjunction with FOXP3, the proportion of Th17-suppressive Tregs based on CD39 expression, as well as the expression of the Treg effector molecule CTLA4. As shown in Figure 4E-G, all Treg subsets were considerably reduced at the 3 month time point. For 4 of the included patients, we also studied the Treg phenotype at 6 months and the decline in frequencies remained low, suggesting that this is a general outcome of costimulation-blockade and not a transient effect (Additional file 1: Figure S1).

### Abatacept reduces synovial T cell function in vitro

In rheumatoid arthritis, affected joints accumulate synovial fluid that often is very cellular with substantial fractions of both effector/memory T cells and Tregs. To assess whether abatacept would also influence T cell functionality at the site of inflammation, we studied the effect of in vitro added abatacept on both synovial T effector and Treg function. By this approach each sample is individually controlled by the wells without abatacept.

**Figure 5:**
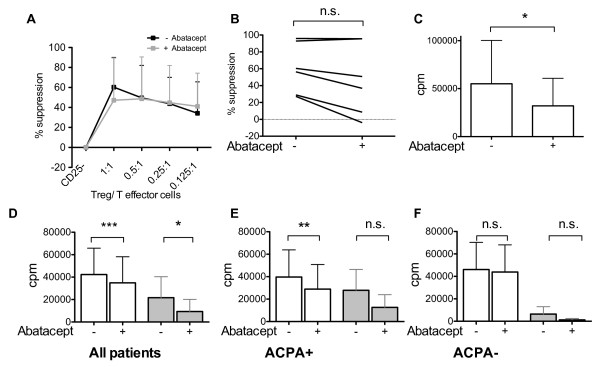
**Abatacept reduces T cell proliferation in vitro. (A**-**C)** SFMC from RA patients (n=6) were sorted into CD4+CD25- T effector cells as well as CD4+CD25++ Treg and were co-cultured with CD3-APC for 6 days in the presence of plate-bound α-CD3, either in the presence or absence of 10 μg/ml abatacept. **(A)** Synovial CD4+CD25- T effector cells were cultured alone and in the presence of different ratios of CD4+CD25++ Treg. The graph depicts the percentage of suppression by CD4+CD25++ Treg in co-culture either in the absence (black line) or the presence of abatacept (grey line). A summary of six experiments is shown and all values are expressed as mean+SD. **(B)** The graph displays the suppression of proliferation at the 1:1 ratio of T effector cells and Tregs. **(C)** Proliferation of CD4+CD25- T effector cells alone was measured by thymidine incorporation in the absence or presence of abatacept (n=6). **(D**-**F)** SFMC from RA patients were stimulated with α-CD3 (white bars) or influenza vaccine (grey bars) in the presence of 10 μg/ml abatacept or a control compound. Proliferation of SFMC was measured by thymidine incorporation following 72 hours (α-CD3) or 6 days (influenza) in culture. All values are expressed as mean+SD. **(D)** All patients are displayed, (α-CD3: n=15 patients; Influenza: n=7). **(E)** Only ACPA-positive patients are displayed, (α-CD3: n=9 patients; Influenza: n=5). **(F)** Only ACPA-negative patients are displayed, (α-CD3: n=6 patients; Influenza: n=2).

First we studied the effect of abatacept on synovial T cells in vitro by investigating suppression in the presence of abatacept. Treg co-culture experiments were performed but no difference in suppressive capacity was seen in cultures with abatacept compared to control cultures (Figure 5A and B). However, we observed a significant reduction of CD25-negative T effector cell proliferation in the presence of abatacept (Figure 5C).

Second, we studied the effects of abatacept on the whole SFMC population using polyclonal and antigen-specific stimulation. As depicted in Figure 5D, abatacept significantly down-regulated the proliferative response following polyclonal (n=15) and antigen-specific (n=7) stimulation. We also observed reduced IFN-γ levels in the culture supernatants of the abatacept-treated cells (data not shown).

The reduced proliferation was confined to the autoantibody-positive fraction of the patients (Figure 5E and F).

## Discussion

Abatacept is a soluble chimeric CTLA4 protein, which binds with high affinity to the B7 molecules CD80 and CD86 expressed on antigen-presenting cells. The outcome of this blockade could be manifold, including changes of activity and lifespan of APCs and limiting the activation and re-activation of CD4+ T cells. In the present study we demonstrate a significant down-regulation of all key T cell effector subsets including Th1, Th2 and Th17 by abatacept. We saw these effects in the ACPA-positive patient subset, but not in the ACPA-negative patients. Further, we could show a general decrease in frequencies of regulatory T cells.

Today it is widely recognized that ACPA-positive and ACPA-negative RA are distinct disease sub-entities as these two phenotypes demonstrate major differences in terms of HLA-association, other genetic and environmental risk factors, clinical disease phenotype and treatment response and probably in molecular pathogenesis as well [17-21].

Clinical response to CTLA4-Ig (abatacept) can be different early in disease as compared to patients with established disease. It has also been shown that sero-conversion (ACPA-positive turning into ACPA-negative) is possible in early RA patients treated with abatacept (ADJUST and AGREE trials) [22,23] suggesting that abatacept may impact on autoantibody formation. However, we could not detect any difference in ACPA levels after 6 months of treatment, but our cohort consists of patients with “long-standing” disease who have failed several biologicals prior to starting with abatacept. Interestingly it was recently published that in a cohort similar to ours (but larger), ACPA status was an independent factor positively correlating to therapy response, where ACPA-positive RA patients responded better to Abatacept treatment than ACPA-negative [11]. Still, abatacept is approved for use in both ACPA-positive and ACPA-negative patients and patients in our study who demonstrated a clinical response towards abatacept were both ACPA-positive and negative. This illustrates how therapeutic progress is possible even though the role of T cells in ACPA-positive vs. ACPA-negative RA is not (fully) understood.

Importantly, our study aimed at dissecting the outcome of costimulation blockade by abatacept on a cell population level. It was not powered to address clinical outcome or identify biomarkers for therapy response. In particular the number of ACPA-negative patients in our cohort was low, reflecting a typical RA cohort of patients with long-standing disease.

To address the question of which cells are affected by abatacept therapy, we studied both peripheral blood samples of patients undergoing therapy as well as synovial fluid cells from disease-active joints where abatacept has been added to the cultures. Recently another study focusing on T helper cells in affected joints was published. Consistent with our data, they showed that CTLA4-Ig leads to reduced levels of proinflammatory cytokines IFN-γ and IL-2 but they also reported increased levels of IL-10 and TGF-β. ACPA status was not reported [24].

In our study we investigated cytokine production representative for different T cell subsets (Th1, Th2, Th17), utilizing PBMCs of abatacept-treated patients by two different methodological approaches. Strikingly, a reduction in Th1, Th2 and Th17 cytokines in ACPA-positive patients was seen, but not in ACPA-negative patients. The observed down-regulation of IFN-γ is supported by a previous study by Cutolo et al. showing that abatacept in vitro can decrease synovial macrophage activation when co-cultured with T cells accompanied with a decreased production of proinflammatory cytokines such as IL-6 and TNF [25]. A few years ago, Buch et al. investigated synovial biopsies from established RA and could demonstrate a decrease of number of B cells after 16 weeks of therapy and reduced mRNA levels for IFN-γ [26]. The ACPA status was not reported in these studies.

We also explored the regulatory T cell compartment (Treg) since it is well established that optimal Treg functionality is dependent on costimulation as antigen-specific Tregs are the most efficient suppressor cells [27]. We have recently shown that synovial Tregs proliferate in vivo [28], suggesting that Tregs in the rheumatic joint interact with their cognate antigens. In the same study, we also showed that biological therapies targeting TNF and IL-6R could lead to increased suppression by synovial Tregs [28]. This is in line with studies performed on Tregs in the circulation of RA patients [29,30]. However, we could not detect increased suppressive capacity, even though the T effector proliferation rate was reduced by abatacept. This observation supports the importance of costimulation for Treg function. Thus, the clinical efficacy seen with abatacept is not due to increased Treg functionality.

The focus of the phenotype study was on natural Tregs expressing the lineage marker FOXP3, and subsets thereof. Overall we found a general reduction of FOXP3+ natural Tregs in the periphery, supporting the data by Alvarez-Quiroga et al, who demonstrated reduced Treg frequencies based on FOXP3 and CD25 expression [31].

## Conclusion

Our study shows that abatacept has a significant impact on T effector functions of the Th1, Th2 and Th17 subsets and that effects were predominantly seen in the ACPA-positive patient subset. Treg frequencies were diminished in the periphery, however no significant changes in function were seen in synovial in vitro co-culture essays. This data gives further evidence that RA has to be seen as a disease with several different sub-entities, and supports the view that ACPA-positive and ACPA-negative patients represent immunologically distinct disease phenotypes, with repercussions for treatment strategies.

## Methods

### Patients and samples

Two cohorts of patients were collected: a first cohort consisted of a total of 29 patients treated with abatacept. Patients had a mean age of 55(18–74), 79% female (n=23), 65% ACPA-positive (n=19). PBMCs and serum from RA patients were collected at month 0, on initiation of abatacept therapy, and after 3 months and 6 months of therapy. Patients were treated with abatacept by intravenous infusion according to baseline weight (< 60 kg, 500 mg; 60–100 kg, 750 mg; and > 100 kg, 1000 mg) on days 1, 15, 29, and then every 4 weeks. Clinical assessment of the patients was performed after 3 and 6 months and the clinical response was evaluated using the European League Against Rheumatism (EULAR) response criteria, based on the disease activity score using the 28 joint count (DAS28) and erythrocyte sedimentation rate. The second cohort consisted of 16 RA and 6 JIA patients, from which Synovial fluid and serum was obtained. (Mean age 51(23–86), 77% female (n=17), 50% ACPA-positive (n=11)). All patients attended the Rheumatology Clinic at Karolinska University Hospital. All RA patients fulfilled the ACR criteria for RA [32].

Peripheral blood and synovial fluid samples prepared by ficoll (Ficoll-Paque Plus, GE Healthcare, Uppsala, Sweden) separation and cryopreserved until use. Serum samples were stored at −70°C until use.

The ethics review board of the Karolinska University Hospital approved this study, and all study subjects gave informed consent according to the declaration of Helsinki.

### Anti-CCP assay (ACPA)

Serum samples from 33 patients were used to determine anti-citrullinated cyclic peptides (anti-CCP) levels using the anti-CCP-2 ELISA kit (Immunoscan RA Mark 2; Euro-Diagnostica, Arnhem, The Netherlands) according to the manufacturer’s instructions.

### Intracellular cytokine staining

Cells from 19 patients collected at 0 and 6 months after abatacept treatment were cultured for 6h or 5 days in complete media (RPMI, HEPES, L-Glutamine, Penicillin, Streptomycin) containing 5% human serum and stimulated with plate-bound α-CD3 monoclonal antibody (2.5 μg/ml, clone OKT-3). All cultures were incubated at 37°C, 5% CO_2_. Supernatants were collected on day 5 and stored at −80°C until use.

In order to prevent produced cytokines from being secreted, 10 μg/ml Brefeldin A (Sigma-Aldrich, Steinheim, Germany) was added to the cultures 4 h prior to harvesting. Extracellular and intracellular cytokine staining was performed using Cytofix/Cytoperm Kit (BD) according to the manufacturer’s instructions. Antibodies: α-CD28 PE (clone: L293, BD), α-CD14 APC Cy7 (clone: MphiP9, BD), α-CD4 PeCy7 (clone: SK3, BD), α-CD3 PB (clone: UCHT1, BD and Biolegend), α-IFN-γ FITC (clone: B27, BD), α-TNF PerCP Cy5.5 (clone: Mab11, Biolegend), α-IL17 Alexa 647 (clone: BL168, Biolegend).

Beriglobin was added in order to prevent unspecific staining and LIVE/DEAD Aqua Dead Cell Stain (Invitrogen) was used to exclude dead cells. The PBMCs were run on a Beckman Coulter CyAn. Analyses were performed with FlowJo software, version 8.1.0 or higher (Treestar Inc.).

### Luminex analysis of cell culture supernatants

Cytokine analysis was performed on the collected supernatant samples from the 5 day cultures using the multiplex assay LEGENDplex (Biolegend, San Diego, CA, USA) and read on a Luminex100™ platform (Bio-Rad, Hercules, CA, USA) with Bio-Rad software. The cytokines analyzed were; interleukin IL-1β (1.9), IL-2 (0.3), IL-3 (4.1), IL-4 (0.6), IL-7 (1.5), IL-9 (0.1), IL-10 (0.2), IL-13 (0.2), IL-17A (0.7), IL-17F (1.6), IL-21 (0.2), IL-22 (4.4), IL-23 (1.2), TNF (0.6) and IFN-γ (0.2). Detection limits for these cytokines/chemokines are indicated in brackets in pg/ml.

### Phenotypic characterization of Tregs

PBMCs taken from 12 patients at 0 and 3 months post-treatment were utilized for phenotypic analyses by flow cytometry. Intranuclear staining of FOXP3 and Helios was performed using FOXP3/Transcription factor staining kit (eBioscience, San Diego, CA, USA) according to the manufacturer’s instructions. Antibodies: α-CD3 Alexa700 (clone: UCHT1, Biolegend), α-CD3 FITC (clone: UCHT1, BD), α-CD4 PE (clone: RPA-T4, BD), α-CD39 FITC (clone: A1, Biolegend), α-CD45RA ECD (clone: 2H4LDH11LDB9, Beckman Coulter), α-Helios Alexa647 (clone: 22F6, Biolegend), α-FOXP3 PB (clone: 206D, Biolegend), α-CTLA4 PE (clone: BNI-3, BD). LIVE/DEAD Near-IR Dead Cell Stain (Invitrogen) was used to exclude dead cells. The PBMCs were run on a Beckman Coulter Gallios (Beckman Coulter, Brea, CA, USA). Analyses were performed with FlowJo software, version 8.1.0 or higher (Treestar Inc., Ashland, OR; USA).

### In vitro study of SFMC in the presence of abatacept

SFMC (n=16) were thawed and stimulated with either plate-bound α-CD3 (1 μg/ml, clone: OKT-3) for 72 hours or with influenza vaccine (Fluvirin vaccine 2001/2002, Evans Vaccines Limited, Liverpool, UK) for 6 days. For Treg suppression assays, SFMC (n=6) were thawed and sorted by flow cytometry into CD3-APC, CD3+4+25- effector T cells and CD3+4+25++ Tregs and co-cultured with plate-bound α-CD3 (0.5 μg/ml, clone OKT-3) for 6 days [28] in presence or absence of 10 μg/ml abatacept. For total SFMC Abatacept or a control compound (Chimeric L6 Bd2.1 IgG) was added at 10 μg/ml (both provided by Bristol-Myers Squibb, Princeton, NJ, USA), which represents the physiological concentration in the blood of patients post-treatment [33]. During the last 15–18 hours of the incubation ^3^H-labelled thymidine (1uCi/well, Perkin Elmer, Boston, MA, USA) was added to the wells and the counts per minute were measured, indicating cell division. Cytokines were measured in supernatants with the CBA Inflammation Kit (Becton Dickinson (BD), San Jose, CA, USA) according to the manufacturer’s instructions and were acquired and analyzed with a FACS calibur (BD).

### Statistical analysis

A nonparametric Wilcoxon signed-rank test was used to compare control and abatacept-treated SFMC cultures, Treg co-cultures, Treg frequencies, and differences between baseline and post-treatment cytokine secretion and cytokine levels. *P* values less than 0.05 were considered statistically significant. Spearman’s rank correlation coefficient was used to analyze the ratio between different combinations of cytokines.

All statistical analyses were performed using Prism 5.0 (GraphPad Software, La Jolla, CA, USA).

## Competing interests

Our Rheumatology unit receives support from a national Swedish initiative for research, patient participation and collaboration with industry concerning inflammatory diseases. This consortium receives financial contributions for infrastructure as well as for distinct research projects from several companies including NovoNordisk, Pfizer, Sobi and BMS. The rheumatology unit also receives support from the Innovative Medicines Initiative BTCure. Also in this consortium, several companies including Astra-Zeneca, NovoNordisk, UCB and BMS participate. Our head of research, professor Lars Klareskog is PI and financially responsible for both these consortia. Consulting fees/honoraria for RvV (<10,000$): Abbott, Bristol-Myers Squibb, Merck, Pfizer, GSK, Roche, UCB Pharma. Grant/research support for RvV: same as above.

## Authors' contributions

JP carried out and analyzed the proliferation study, flow cytometric study and luminex, participated in its design and drafted the manuscript. JH carried out phenotypic and functional assessment of Treg studies and participated in drafting the manuscript. SR and KM participated in the proliferation study and the design. RvV was responsible for patients’ inclusion and interpretation of clinical data. VM conceived the study, and participated in its design and coordination and helped to draft the manuscript. All authors read and approved the final manuscript.

## Supplementary Material

Additional file 1: Figure S1 Treg frequency is diminished. PBMCs from baseline, 3 months and 6 months following treatment (n=4) were obtained and multicolor flow cytometry was performed. A general reduction in frequency of several Treg associated markers could be seen (A-F). (A) The graph displays CD4+FOXP3+ Treg, (B) Helios+ T cells, (C) CD4+CD39+ T cells, (D) CD45RA+FOXP3+ Treg, (E) CTLA4+ FOXP3+Treg, and (F) CD39+FOXP3+ Treg. Click here for file
