# Supplementary figures and images for: CTLA4-Ig (abatacept) therapy modulates T cell effector functions in autoantibody-positive rheumatoid arthritis patients
Source: BMC Immunol. 2013 Aug 5;14:34. doi: 10.1186/1471-2172-14-34 (PMC3750242; doi:10.1186/1471-2172-14-34)

**A**

FOXP3+

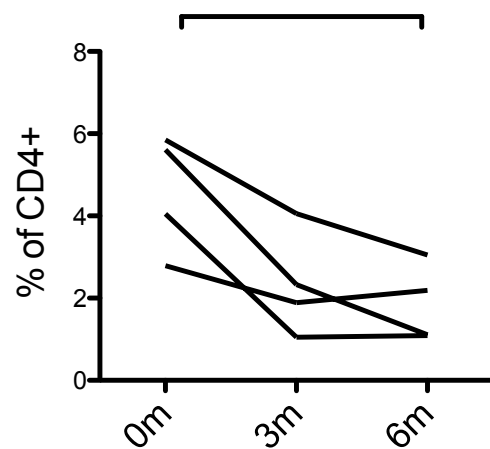**B**

Helios

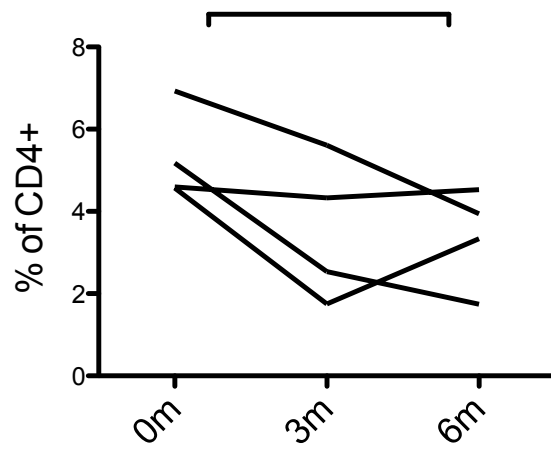**C**

CD39

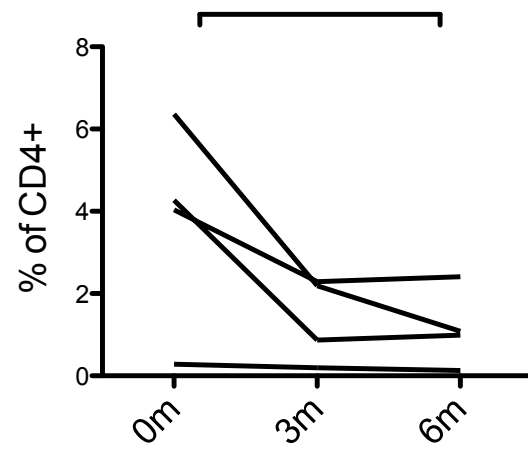**D**

FOXP3+

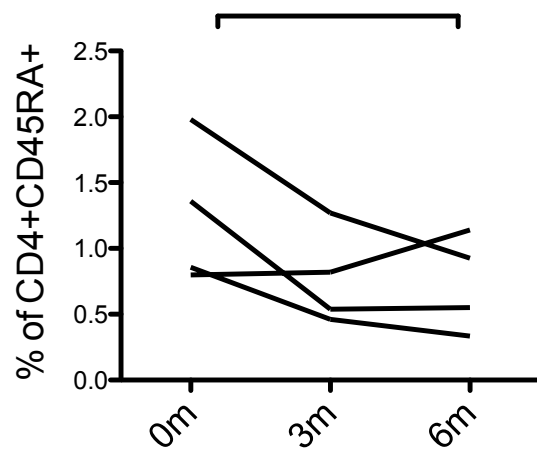**E**

FOXP3+

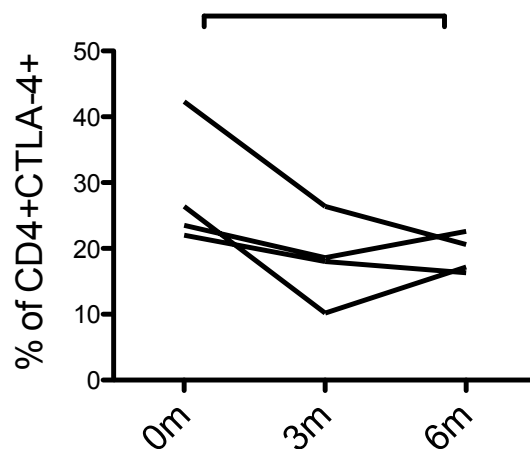**F**

FOXP3+

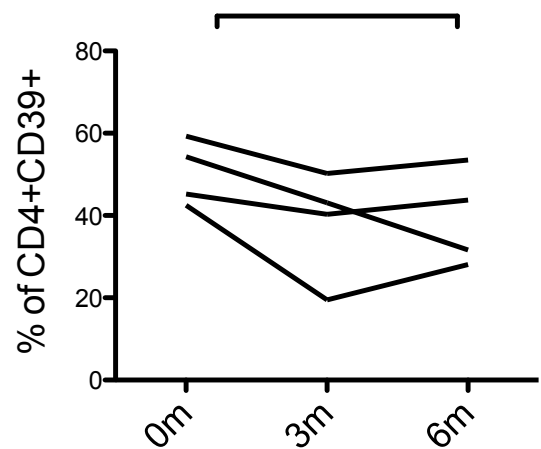

Supplement: Additional file 1: Figure S1 — Treg frequency is diminished. PBMCs from baseline, 3 months and 6 months following treatment (n=4) were obtained and multicolor flow cytometry was performed. A general reduction in frequency of several Treg associated markers could be seen (A-F). (A) The graph displays CD4+FOXP3+ Treg, (B) Helios+ T cells, (C) CD4+CD39+ T cells, (D) CD45RA+FOXP3+ Treg, (E) CTLA4+ FOXP3+Treg, and (F) CD39+FOXP3+ Treg. [file 1471-2172-14-34-S1.pdf]
